# Genome sequence and emended description of *Leisingera nanhaiensis* strain DSM 24252^T^ isolated from marine sediment

**DOI:** 10.4056/sigs.3828824

**Published:** 2014-01-25

**Authors:** Sven Breider, Hazuki Teshima, Jörn Petersen, Olga Chertkov, Hajnalka Dalingault, Amy Chen, Amrita Pati, Natalia Ivanova, Alla Lapidus, Lynne A. Goodwin, Patrick Chain, John C. Detter, Manfred Rohde, Brian J. Tindall, Nikos C. Kyrpides, Tanja Woyke, Meinhard Simon, Markus Göker, Hans-Peter Klenk, Thorsten Brinkhoff

**Affiliations:** 1 Institute for Chemistry and Biology of the Marine Environment, University of Oldenburg, Oldenburg, Germany.; 2 Los Alamos National Laboratory, Bioscience Division, Los Alamos, New Mexico, USA; 3 Leibniz Institute DSMZ - German Collection of Microorganisms and Cell Cultures, Braunschweig, Germany; 4 Biological Data Management and Technology Center, Lawrence Berkeley National Laboratory, Berkeley, California, USA; 5 DOE Joint Genome Institute, Walnut Creek, California, USA; 6 HZI – Helmholtz Centre for Infection Research, Braunschweig, Germany

**Keywords:** Marine, motile, facultative anaerobe, methylated compounds, *Rhodobacteraceae*, *Roseobacter* clade

## Abstract

*Leisingera nanhaiensis* DSM 24252^T^ is a Gram-negative, motile, rod-shaped marine *Alphaproteobacterium*, isolated from sandy marine sediments. Here we present the non-contiguous genome sequence and annotation together with a summary of the organism's phenotypic features. The 4,948,550 bp long genome with its 4,832 protein-coding and 64 RNA genes consists of one chromosome and six extrachromosomal elements with lengths of 236 kb, 92 kb, 61 kb, 58 kb, 56 kb, and 35 kb, respectively. The analysis of the genome showed that DSM 24252^T^ possesses all genes necessary for dissimilatory nitrite reduction, and the strain was shown to be facultatively anaerobic, a deviation from the original description that calls for an emendation of the species. Also present in the genome are genes coding for a putative prophage, for gene-transfer agents and for the utilization of methylated amines. Phylogenetic analysis and intergenomic distances indicate that *L. nanhaiensis* might not belong to the genus *Leisingera*.

## Introduction

The genus *Leisingera* was proposed by Schaefer *et al.* in 2002 [1] and belongs to the family *Rhodobacteraceae* within the class *Alphaproteobacteria*. The genus currently consists of three species with validly published names, with *Leisingera methylohalidivorans* as the type species. The genus was named in honor of Thomas Leisinger on the occasion of his retirement and for his contributions to our understanding of the biochemistry of bacterial methyl-halide metabolism. NH52F^T^ (= DSM 24252^T^ = LMG 24841^T^ = ATCC BAA-92^T^) is the type strain of *L. nanhaiensis* and was isolated from marine sandy sediment taken from the South Chinese Sea [2]. The species name is referring to Nanhai, the Chinese name for the South China Sea. The other two *Leisingera* species were isolated from seawater and a marine electroactive biofilm, respectively [1,3]. All three *Leisingera* species are able to grow on methylated amines as the sole N source [4] and, at least for *L. methylohalidivorans*, the ability to grow on methyl halides as sole carbon source was described [1].

Here we present a summary classification and features of *L. nanhaiensis* DSM 24252^T^, together with the description of the non-contiguous genomic sequence and annotation.

## Classification and features

### 16S rDNA analysis

A representative genomic 16S rDNA sequence of *L. nanhaiensis* DSM 24252^T^ was compared with the Greengenes database for determining the weighted relative frequencies of taxa and (truncated) keywords as previously described [5]. The most frequently occurring genera were *Phaeobacter* (51.0%), *Roseobacter* (20.2%), *Silicibacter* (7.6%), *Leisingera* (5.5%) and *Nautella* (3.9%) (75 hits in total). Regarding the four hits to sequences from other species of the genus, the average identity within HSPs was 96.7%, whereas the average coverage by HSPs was 99.4%. Among all other species, the one yielding the highest score was *L. methylohalidivorans* (NR_025637), which corresponded to an identity of 96.8% and an HSP coverage of 100.1%. [Note that the Greengenes database uses the INSDC (= EMBL/NCBI/DDBJ) annotation, which is not an authoritative source for nomenclature or classification.] The highest-scoring environmental sequence was AJ296158 (Greengenes short name 'Spain:Galicia isolate str. PP-154'), which showed an identity of 96.3% and an HSP coverage of 100.0%. The most frequently occurring keywords within the labels of all environmental samples that yielded hits were 'microbi' (7.7%), 'marin' (7.3%), 'water' (7.0%), 'coastal' (6.6%) and 'effect' (6.6%) (168 hits in total). Environmental samples that yielded hits of a higher score than the highest scoring species were not found, indicating that this species is rarely detected in the environment.

**Figure 1:**
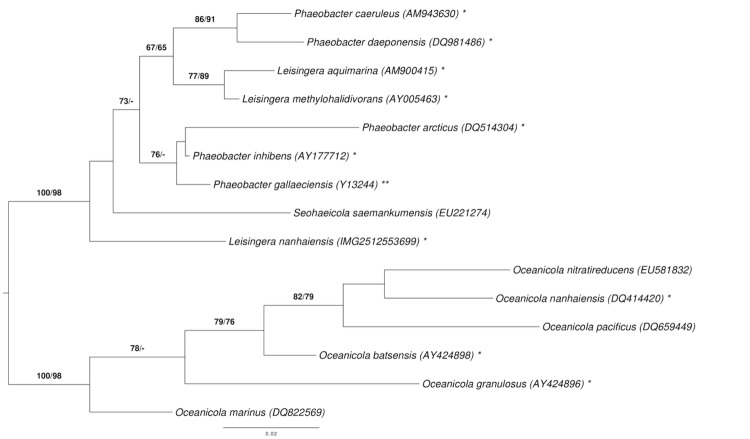
Phylogenetic tree highlighting the position of *L. nanhaiensis* relative to the type strains of the other species within the genus *Leisingera* and the neighboring genera *Phaeobacter*, *Oceanicola*, and *Seohaeicola* [1-3,6-16]. The tree was inferred from 1,385 aligned characters of the 16S rRNA gene sequence under the maximum likelihood (ML) criterion as previously described [5]. *Oceanicola* spp. were included in the dataset for use as outgroup taxa. The branches are scaled in terms of the expected number of substitutions per site. Numbers adjacent to the branches are support values from 1,000 ML bootstrap replicates (left) and from 1,000 maximum-parsimony bootstrap replicates (right) if larger than 60% [5]. Lineages with type strain genome sequencing projects registered in GOLD [17] are labeled with one asterisk, those also listed as 'Complete and Published' with two asterisks [10,18-23].

Figure 1 shows the phylogenetic neighborhood of *L. nanhaiensis* in a tree based on 16S rRNA gene sequences. The sequences of the two identical 16S rDNA copies in the genome do not differ from the previously published 16S rRNA gene sequence (FJ232451).

Our phylogenetic analysis (Figure 1, Table 1) indicates that *L. nanhaiensis* is not particularly closely affiliated with the other *Leisingera* species. BLAST results against the NCBI database with the 1,429 bp long 16S rRNA gene sequence showed 97% similarity to *L. methylohalidivorans* strain MB2, *Phaeobacter gallaeciensis* DSM 17395 and *P. gallaeciensis* 2.10 (see also the Greengenes analysis described above). Thus a reclassification of *L. nanhaiensis* might be appropriate, but should probably be postponed until more genome sequences from the relevant genera are available, as the 16S rRNA gene trees are only partially resolved (Figure 1). A preliminary phylogenomic analysis is given below.

**Table 1:** Classification and general features of *L. nanhaiensis* DSM 24252^T^ according to the MIGS recommendations [24] published by the Genome Standards Consortium [25].

**MIGS ID**	**Property**	**Term**	**Evidence code**
		Domain *Bacteria*	TAS [26]
		Phylum *Proteobacteria*	TAS [27,28]
		Class *Alphaproteobacteria*	TAS [28,29]
	Current classification	Order *Rhodobacterales*	TAS [28,30]
		Family *Rhodobacteraceae*	TAS [28,31]
		Genus *Leisingera*	TAS [1,3,32]
		Species *Leisingera nanhaiensis*	TAS [2]
MIGS-7	Subspecific genetic lineage (strain)	NH52F^T^	TAS [2]
MIGS-12	Reference for biomaterial	Sun *et al* 2010.	TAS [2]
	Gram stain	Gram-negative	TAS [2]
	Cell shape	Rod-shaped	TAS [2]
	Motility	Yes	TAS [2]
	Sporulation	Not reported	
MIGS-6.1	Temperature range	4-37 °C	TAS [2]
MIGS-6.1	Optimum temperature	25°C	TAS [2]
MIGS-6.3	Salinity	halophile	TAS [2]
MIGS-22	Relationship to oxygen	facultatively anaerobe	IDA
	Carbon source	complex substrates, betaine, methionine	TAS [2]
	Energy metabolism	Not reported	
MIGS-6	Habitat	sea water, sediment, sand	TAS [2]
MIGS-6.2	pH	pH 6.0–9.3 (optimal, pH 7-8.5)	TAS [2]
MIGS-15	Biotic relationship	free living	TAS [2]
MIGS-14	Known pathogenicity	Not reported	
MIGS-16	Specific host	Not reported	
MIGS-18	Health status of host	Not reported	
	Biosafety level	1	TAS [33]
MIGS-19	Trophic level	Not reported	
MIGS-23	Isolation	sandy sediments	TAS [2]
MIGS-4	Geographic location	South China Sea	TAS [2]
MIGS-5	Time of sample collection	before 2009	NAS
MIGS-4.1	Latitude	15.55	TAS [2]
MIGS-4.2	Longitude	114.49	TAS [2]
MIGS-4.3	Depth	157 m	NAS
MIGS-4.4	Altitude	Not reported	

Evidence codes – TAS: Traceable Author Statement (i.e., a direct report exists in the literature); NAS: Non-traceable Author Statement (i.e., not directly observed for the living, isolated sample, but based on a generally accepted property for the species, or anecdotal evidence); IDA: Inferred from Direct Assay. Evidence codes are from the Gene Ontology project [34].

### Morphology and physiology 

**Figure 2:**
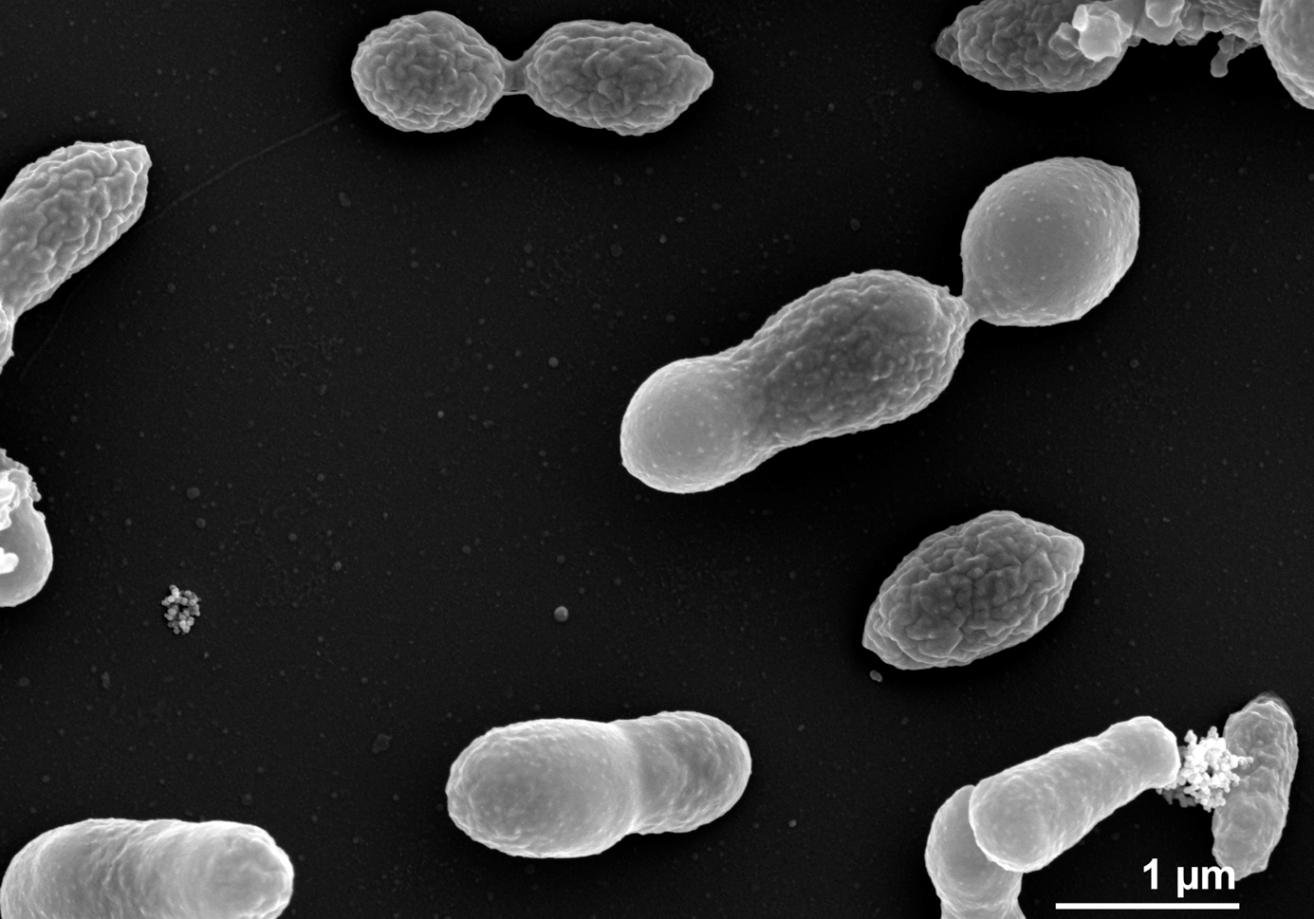
Scanning electron micrograph of *L. nanhaiensis* DSM 24252^T^

*L. nanhaiensis* NH52F^T^ was originally described as an aerobe [2], but as genes for the dissimilatory reduction of nitrite could be found in the genome of DSM 24252^T^ (see “Insights into the genome”) the organisms has the genetic potential to be a facultative anaerobe. Cells of strain NH52F^T^ are Gram-negative, motile rods, 0.62 – 0.8 x 1.6 – 2.96 µm in size [2]. Figure 2 shows a scanning-electron micrograph of *L. nanhaiensis* DSM 24252^T^. NaCl is essential for growth, which occurs from 0.6% to 6.0% NaCl with an optimum between 1% and 4% [2]. The temperature range is 4°C – 37°C (optimum 25°C) and the pH range is 6 – 9.3 (optimum 7 – 8.5). Growth only occurs on complex substrates such as yeast extract, tryptone and peptone from potatoes, as well as betaine and methionine [2]. The color of the colonies grown on complex medium (M2 agar medium) is beige. The type strain is susceptible to a broad spectrum of antibiotics listed in [2].

### Chemotaxonomy

The main cellular fatty acids of strain NH52F^T^ are (>1% of total fatty acids) C_18:1_ *_ω_*_7c_, an unknown fatty acid (eqivalent chain-length of 11.799), C_16:0 2-OH_, C_10:0 3-OH_, C_16:0_, 11-methyl C_18:1_*_ω_*_7c_ and C_12:0 3-OH_. The major polar lipids are phosphatidylglycerol, phosphatidylethanolamine, an unidentified phospholipid, an unidentified lipid and an aminolipid [2].

## Genome sequencing and annotation

### Genome project history

This organism was selected for sequencing on the basis of the DOE Joint Genome Institute Community Sequencing Program 2010, CSP 441: “Whole genome type strain sequences of the genera *Phaeobacter* and *Leisingera* – a monophyletic group of physiologically highly diverse organisms”. The genome project is deposited in the Genomes On Line Database [17] and the complete genome sequence is deposited in GenBank. Sequencing, finishing and annotation were performed by the DOE Joint Genome Institute (JGI) using state-of-the-art sequencing technology [35]. A summary of the project information is shown in Table 2.

**Table 2:** Genome sequencing project information

**MIGS ID**	**Property**	**Term**
MIGS-31	Finishing quality	Non-contiguous finished
MIGS-28	Libraries used	Two Illumina paired-end libraries (270 bp and 8 kb insert size)
MIGS-29	Sequencing platforms	Illumina GAii
MIGS-31.2	Sequencing coverage	711 × Illumina
MIGS-30	Assemblers	Allpaths version r39750, Velvet 1.1.05, phrap version SPS - 4.24
MIGS-32	Gene calling method	Prodigal 1.4, GenePRIMP
	INSDC ID	AXBG00000000
	GenBank Date of Release	August 23, 2013
	GOLD ID	Gi10857
	NCBI project ID	78295
	Database: IMG	2512047087
MIGS-13	Source material identifier	DSM 24252

### Growth conditions and DNA isolation

A culture of DSM 24252^T^ was grown in DSMZ medium 514 (Bacto Marine Broth) [36] at 28°C. gDNA was purified using Jetflex Genomic DNA Purification Kit (GENOMED 600100) following the directions provided by the supplier but modified by the use of a 40 min incubation time. The purity, quality and size of the bulk gDNA preparation were assessed by JGI according to DOE-JGI guidelines. DNA is available through the DNA Bank Network [37].

### Genome sequencing and assembly

The draft genome sequence was generated using Illumina data [38]. For this genome, we constructed and sequenced an Illumina short-insert paired-end library with an average insert size of 270 bp which generated 13,912,778 reads and an Illumina long-insert paired-end library with an average insert size of 7,381 ± 2,326 bp which generated 9,786,858 reads totaling 3,555 Mbp of data (Feng Chen, unpublished data). All general aspects of library construction and sequencing can be found at the JGI web site [39]. The initial draft assembly contained 43 contigs in 14 scaffolds. The initial draft data was assembled with Allpaths and the consensus was computationally shredded into 10 kbp overlapping fake reads (shreds). The Illumina draft data was also assembled with Velvet [40], and the consensus sequences were computationally shredded into 1.5 kbp overlapping fake reads (shreds). The Illumina draft data was assembled again with Velvet using the shreds from the first Velvet assembly to guide the next assembly. The consensus from the second Velvet assembly was shredded into 1.5 kbp overlapping fake reads. The fake reads from the Allpaths [41] assembly and both Velvet assemblies and a subset of the Illumina CLIP paired-end reads were assembled using parallel phrap (High Performance Software, LLC). Possible mis-assemblies were corrected with manual editing in Consed [42-44]. Gap closure was accomplished using repeat resolution software (Wei Gu, unpublished data), and sequencing of bridging PCR fragments with Sanger technology. One round of manual/wet lab finishing was completed. A total of 43 additional sequencing reactions were completed to close gaps and to raise the quality of the final sequence. The estimated size of the genome is 5 Mb and the final assembly is based on 3,555 Mbp of Illumina draft data, which provides an average 711 × coverage of the genome.

### Genome annotation

Genes were identified using Prodigal [45] as part of the JGI genome annotation pipeline [46], followed by a round of manual curation using the JGI GenePRIMP pipeline [47]. The predicted CDSs were translated and used to search the National Center for Biotechnology Information (NCBI) nonredundant database, UniProt, TIGR-Fam, Pfam, PRIAM, KEGG, COG, and InterPro databases. Additional gene-prediction analysis and functional annotation was performed within the Integrated Microbial Genomes - Expert Review (IMG-ER) platform [48].

## Genome properties

The genome consists of seven scaffolds with a total length of 4,948,550 bp and a G+C content of 60.7% (Figures 3a, 3b, 3c, 3d, 3e, 3f and Figure 3g, Table 3). The scaffolds correspond to a chromosome 4,411,177 bp in length and six extrachromosomal elements. Of the 4,896 genes predicted, 4,832 were protein-coding genes and 64 RNAs. The majority of the protein-coding genes (81.1%) were assigned a putative function while the remaining ones were annotated as hypothetical proteins. The distribution of genes into COGs functional categories is presented in Table 4.

**Figure 3a:**
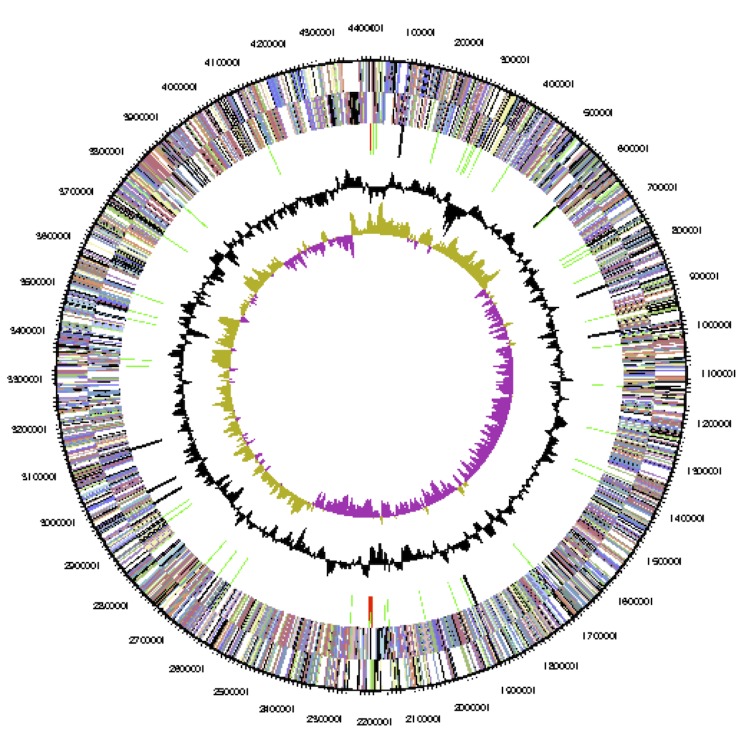
Graphical map of the chromosome (cNanh_4411). From outside to the center: genes on forward strand (color by COG categories), genes on reverse strand (color by COG categories), RNA genes (tRNAs green, rRNAs red, other RNAs black), GC content, GC skew.

**Figure 3b:**
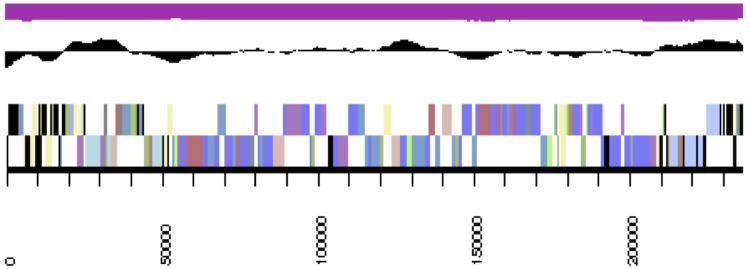
Graphical map of the extrachromosomal element pNanh_A236. From bottom to top: genes on forward strand (color by COG categories), genes on reverse strand (color by COG categories), RNA genes (tRNAs green, rRNAs red, other RNAs black), GC content, GC skew.

**Figure 3c:**
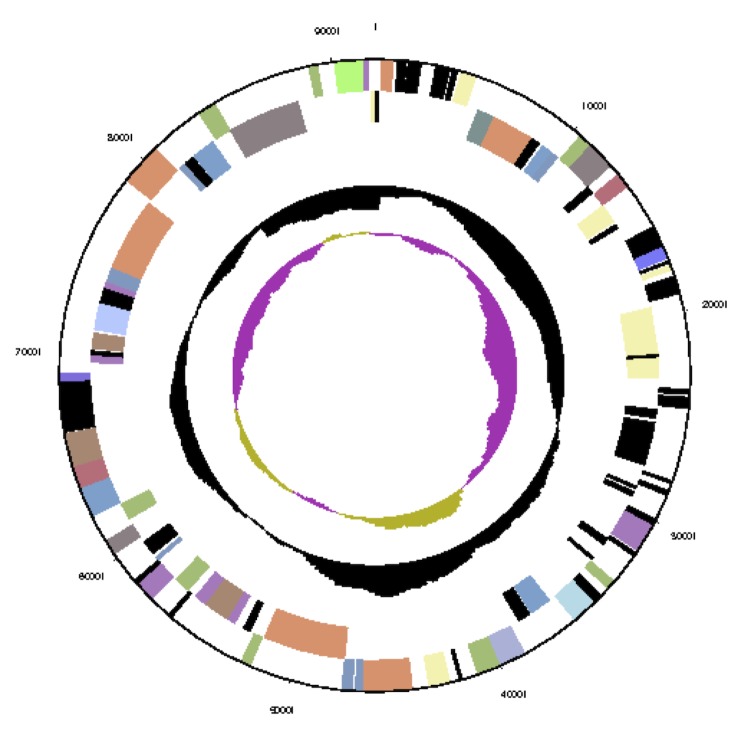
Graphical maps of the extrachromosomal element pNanh_B92. From outside to the center: genes on forward strand (color by COG categories), genes on reverse strand (color by COG categories), RNA genes (tRNAs green, rRNAs red, other RNAs black), GC content, GC skew.

**Figure 3d:**
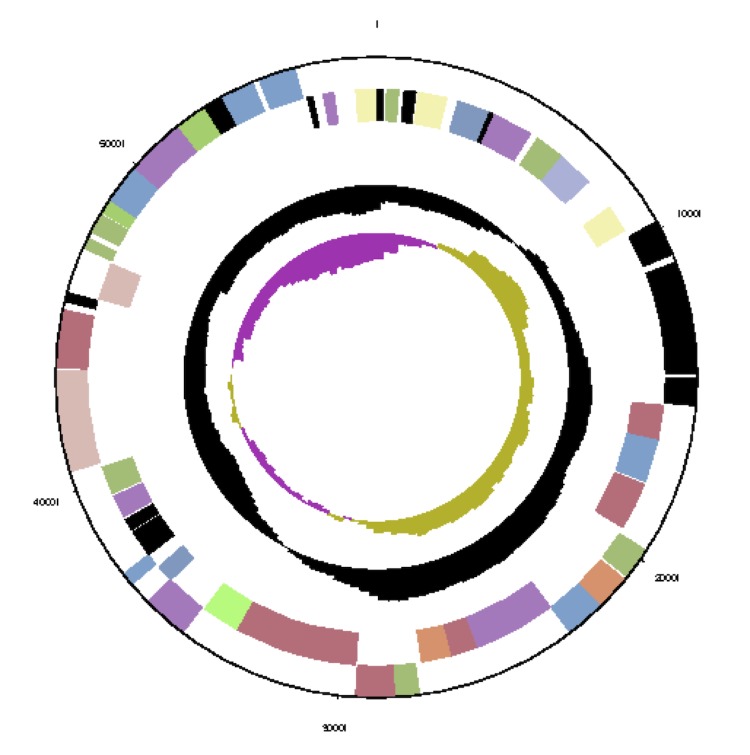
Graphical maps of the extrachromosomal element pNanh_D58. From outside to the center: genes on forward strand (color by COG categories), genes on reverse strand (color by COG categories), RNA genes (tRNAs green, rRNAs red, other RNAs black), GC content, GC skew.

**Figure 3e:**
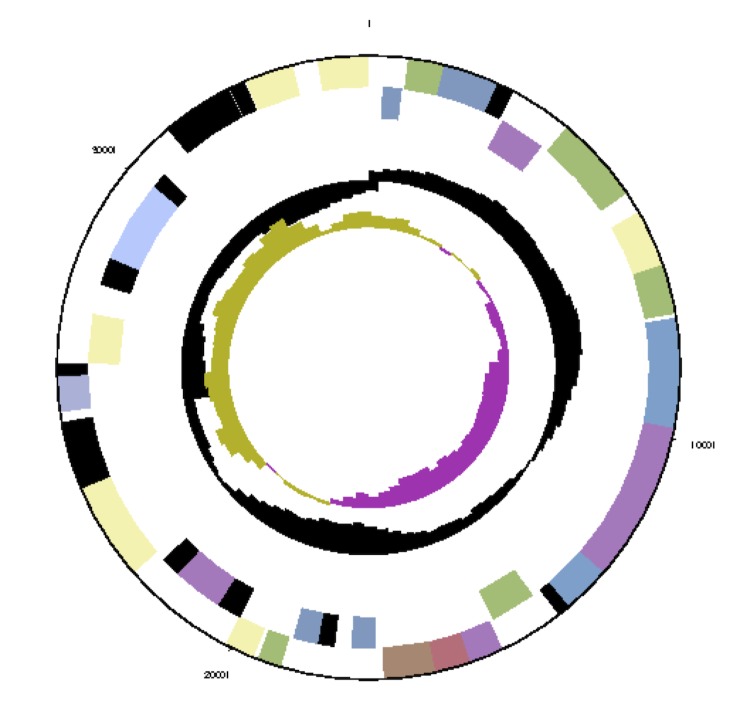
Graphical maps of the extrachromosomal element pNanh_F35. From outside to the center: genes on forward strand (color by COG categories), genes on reverse strand (color by COG categories), RNA genes (tRNAs green, rRNAs red, other RNAs black), GC content, GC skew.

**Figure 3f:**
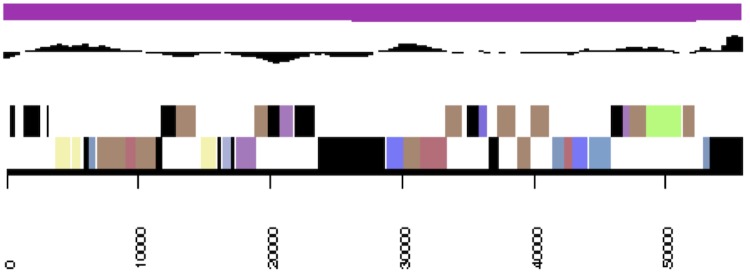
Graphical map of the extrachromosomal element pNanh_E56. From left to right: genes on forward strand (color by COG categories), genes on reverse strand (color by COG categories), RNA genes (tRNAs green, rRNAs red, other RNAs black), GC content, GC skew.

**Figure 3g:**
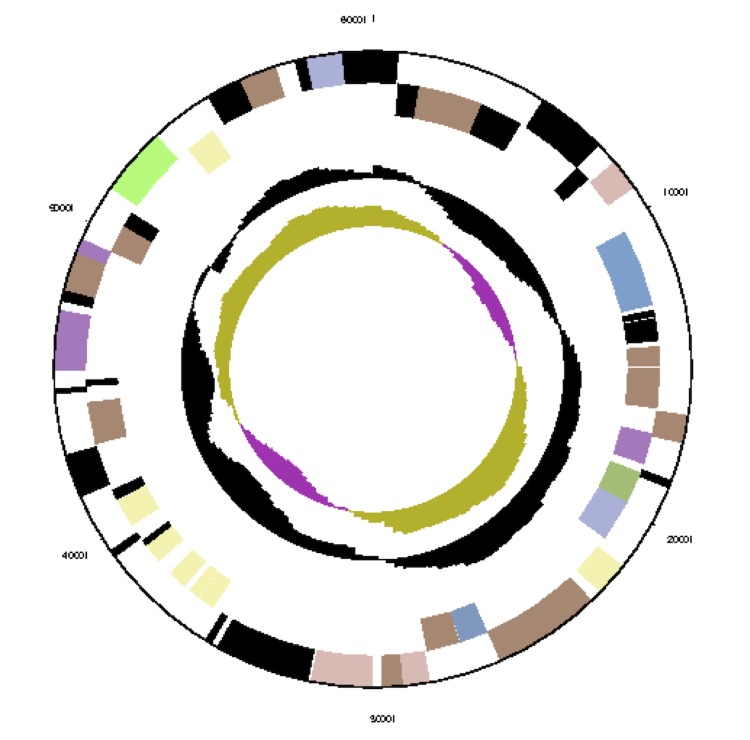
Graphical maps of the extrachromosomal element pNanh_C61. From outside to the center: genes on forward strand (color by COG categories), genes on reverse strand (color by COG categories), RNA genes (tRNAs green, rRNAs red, other RNAs black), GC content, GC skew.

**Table 3:** Genome Statistics

**Attribute**	**Number**	**% of Total**
Genome size (bp)	4,948,550	100.00
DNA coding region (bp)	4,430,400	89.53
DNA G+C content (bp)	3,005,972	60.74
Number of replicons	7	
Extrachromosomal elements	6	
Total genes	4,896	100.00
RNA genes	64	1.31
rRNA operons	2	
tRNA genes	49	1.00
Protein-coding genes	4,832	98.69
Pseudo genes	0	0.00
Genes with function prediction	3,970	81.09
Genes in paralog clusters	3,848	78.59
Genes assigned to COGs	3,813	77.88
Genes assigned Pfam domains	4,051	82.74
Genes with signal peptides	426	8.70
Genes with transmembrane helices	1,059	21.63

**Table 4:** Number of genes associated with the general COG functional categories

**Code**	**Value**	**%age**	**Description**
J	172	4.11	Translation, ribosomal structure and biogenesis
A	1	0.02	RNA processing and modification
K	299	7.14	Transcription
L	249	5.95	Replication, recombination and repair
B	4	0.10	Chromatin structure and dynamics
D	39	0.93	Cell cycle control, cell division, chromosome partitioning
Y	0	0	Nuclear structure
V	63	1.50	Defense mechanisms
T	113	2.70	Signal transduction mechanisms
M	199	4.75	Cell wall/membrane/envelope biogenesis
N	33	0.79	Cell motility
Z	0	0	Cytoskeleton
W	0	0	Extracellular structures
U	77	1.84	Intracellular trafficking, secretion, and vesicular transport
O	147	3.51	Posttranslational modification, protein turnover, chaperones
C	288	6.88	Energy production and conversion
G	209	4.99	Carbohydrate transport and metabolism
E	523	12.49	Amino acid transport and metabolism
F	88	2.10	Nucleotide transport and metabolism
H	167	3.99	Coenzyme transport and metabolism
I	247	5.9	Lipid transport and metabolism
P	217	5.18	Inorganic ion transport and metabolism
Q	159	3.8	Secondary metabolites biosynthesis, transport and catabolism
R	509	12.16	General function prediction only
S	384	9.17	Function unknown
	1,083	22.12	Not in COGs
-	**4,187**	-	Total

## Insights into the genome

The replication-initiation systems identified on the scaffolds were as follows: cNanh_4411, dnaA, repB-I and rep ABC-2; pNanh_A236, repABC-5; pNanh_B92, dnaA-like and repA-d; pNanh_D58, repABC-9; pNanh_F35, repA-a; pNanha_E56, repA-b and repA-c; pNanh_C61, repA-I. This justifies the interpretation of cNanh_4411 as circular chromosome and of the other scaffolds as circular extrachromosomal elements [49,50].

Genome analysis of *L. nanhaiensis* DSM 24252^T^ also revealed the genes for the utilization of methylated amines (MAs). The key genes from the proposed pathway of MA metabolism code for the enzymes trimethylamine monooxygenase (*tmm*) and gammaglutamylmethylamide synthetase (*gmaS*). The trimethylamine monooxygenase is a flavin-dependent enzyme, recently identified by Chen *et al*. [51]. Comparison of a previously published sequence for a trimethylamine monooxygenase gene in *L. nanhaiensis* DSM 24252^T^ from Chen [4] (GenBank accession number JN797867) showed 99% sequence similarity to the gene of a predicted flavoprotein involved in K+ transport in the genome of DSM 24252^T^ (Nanh_04177). Comparison of the *gmaS* sequence (JN797857) with the genome showed also a 99% sequence similarity to a glutamine synthetase, type III (IMG term: gamma-glutamylmethylamide synthetase, EC 6.3.4.12) (Nanh_04141). These genes give *L. nanhaiensis* the potential to utilize MAs as alternative nitrogen sources [4].

Interestingly, the genes *tmm* and *gmaS* of *L. nanhaiensis* DSM 24252^T^ do not cluster with the corresponding genes of the other *Leisingera* species in phylogenetic trees calculated for these genes. The Tmm sequence (~255 amino acids) clusters with *Ruegeria pomeroyi* and *Roseobacter denitrificans*, the sequence for GmaS (~264 amino acids) with *Ruegeria atlantica* and *Roseobacter sp.* AzwK-3b [4].

Strain DSM 24252^T^ encodes a gene transfer agent (GTA), a virus-like particle that mediates transfer of genomic DNA between prokaryotes without negative effects on the host cell [52]. The GTA cluster has a length of ~17 kb (Nanh_00247-Nanh_00229) and shows structural similarities to GTAs of other *Rhodobacterales* species, e.g. *Phaeobacter inhibens* 2.10 and *P. inhibens* DSM 17395 [53]. (Note that the species affiliation of *P. gallaeciensis* and *P. inhibens* strains had recently been reassessed, resulting in the assignment of the alleged *P. gallaeciensis* type-strain deposit DSM 17395 to *P. inhibens* [54].) Strain DSM 24252^T^ also harbors a putative prophage (Nanh_4518 - Nanh_4531).

We found all genes necessary for dissimilatory nitrite reduction, including the cluster for nitrite reductase (Nanh_03376 – Nanh_03386), the cluster for nitric oxide reductase (Nanh_03387 - Nanh_03394), and the cluster for nitrous oxide reductase (Nanh_01753 - Nanh_01761). *L. nanhaiensis* was described as an aerobic bacterium, but only the reduction of nitrate was tested in the original description of this organism [2]. Based on the genomic information we tested strain DSM 24252^T^ for dissimilatory reduction of nitrite, by using anaerobic marine basal medium according to Cypionka and Pfennig [55] supplemented with nitrite (5mM) and methionine (1mM). Reduction of nitrite was tested photometrically at 545nm after two weeks, using the Griess reaction [56].

The results showed that strain DSM 24252^T^ is able to reduce nitrite under anoxic conditions, demonstrating that it is a facultatively anaerobic organism. Below we propose an according emendation of the species description.

Interestingly, the same was found recently for *Phaeobacter inhibens* T5^T^ [21], which also was initially only tested for the reduction of nitrate and thus described as a strictly aerobic bacterium [32]. A test for nitrite reduction showed, however, that *P. inhibens* is in fact a facultatively anaerobic bacterium [21]. Based on these findings, we suggest that anaerobic growth of roseobacters should not only be tested with nitrate, but also with nitrite.

As indicated by the 16S rRNA gene sequence analysis (Figure 1), the classification of *L. nanhaiensis* might need to be reconsidered. We conducted a preliminary phylogenomic analysis using GGDC [57-59] and the draft genomes of the type strains of the other *Leisingera* and *Phaeobacter* species. The results shown in Table 5 indicate that the DNA-DNA hybridization (DDH) similarities calculated *in silico* of *L. nanhaiensis* to *Phaeobacter* species are, on average, not smaller than those to other *Leisingera* species. The highest value was obtained for *P. arcticus*.

**Table 5:** DDH similarities between *L. nanhaiensis* DSM 24252^T^ and the other *Leisingera* and *Phaeobacter* species (with genome-sequenced type strains)^†^

**Reference species**	**formula 1**	**formula 2**	**formula 3**
*L. aquamarina* (AXBE00000000)	14.50±3.11	19.20±2.28	14.70±2.65
*L. methylohalidivorans* (CP006773, CP006774, CP006775)	14.50±3.11	19.20±2.29	14.60±2.64
*P. arcticus* (AXBF00000000)	14.60±3.12	22.90±2.37	14.80±2.66
*P. caeruleus* (AXBI00000000)	14.50±3.11	19.40±2.29	14.60±2.65
*P. daeponensis* (AXBD00000000)	14.70±3.13	19.60±2.30	14.80±2.66
*P. gallaeciensis* (AOQA01000000)	13.80±3.06	20.20±2.31	14.00±2.61
*P. inhibens* (AXBB00000000)	13.90±3.06	19.50±2.29	14.10±2.61

^†^Calculated *in silico* with the GGDC server version 2.0 [59]. The standard deviations indicate the inherent uncertainty in estimating DDH values from intergenomic distances based on models derived from empirical test data sets (which are always limited in size); see [59] for details. The distance formulas are explained in [57]. The numbers in parentheses are GenBank accession numbers identifying the underlying genome sequences. The accession number of *L. nanhaiensis* is AXBG00000000.

### Plasmids

Genome sequencing of *L. nanhaiensis* DSM 24252^T^ reveals the presence of six plasmids with sizes between 35 kb and 236 kb (Table 6). The circular conformation of the chromosome and four extrachromosomal elements has been experimentally validated. The four larger plasmids contain characteristic replication modules of the RepABC-, DnaA-like and RepA-type comprising a replicase as well as the *parAB* partitioning operon [60]. The respective replicases that mediate the initiation of replication are designated according to the established plasmid classification scheme [61]. The different numbering of, e.g., the replicases RepC-5 and RepC-9 from RepABC-type plasmids corresponds to specific plasmid compatibility groups that are required for a stable coexistence of the replicons within the same cell [62]. The two small replicons pNanh_E56 and pNanh_F35 contain solitary RepA-IV type replicases without a partitioning module. This distribution may correspond with a higher plasmid copy number within the cell, thus assuring the replicon maintenance in the daughter cells after cell division. The additional RepA-IV type replicase that is located on the DnaA-like I plasmid pNanh_B92 may originate from a fusion event of two formerly independent plasmids.

**Table 6:** General genomic features of the chromosome and extrachromosomal replicons from *L. nanhaiensis* strain DSM 24252^T^.^*^

**Replicon**	**Scaffold**	**Replicase**	**Length** (bp)	**GC** (%)	**Topology**	**No. Genes^#^**
Chromosome	1	DnaA	4,411,177	61	circular	4,358
pNanh_A236	2	RepC-5	236,302	61	linear*	236
pNanh_B92	3	DnaA-like I RepA-IVc	92,007	58	circular	101
pNanh_C61	4	RepA-I	60,519	63	circular	58
pNanh_D58	5	RepC-9	57,777	60	circular	57
pNanh_E56	6	RepA-IVb	55,854	59	linear*	45
pNanh_F35	7	RepA-IVa	34,914	62	circular	41

*circularity not experimentally validated; ^#^deduced from automatic annotation.

The locus tags of all replicases, plasmid stability modules and the large *virB4* and *virD4* genes of type IV secretion systems are presented in Table 7. The largest plasmid pNanh_A236 contains a post-segregational killing system (PSK), consisting of a typical operon with two small genes encoding a stable toxin and an unstable antitoxin [63]. Moreover, this RepABC-type plasmid also contains a complete type IV secretion system including the *virB* operon for the formation of a transmembrane channel. The relaxase VirD2, which is required for the strand-specific DNA nicking at the origin of transfer (*oriT*), and the coupling protein VirD4 support the presence of a functional conjugation system on this plasmid [64,65]. The presence of the highly conserved chromosomal genes *virD2* (Nanh_3787) and *virD4* (Nanh_3786), representing the relaxase and coupling protein, respectively, is noteworthy. However, the presence of two genes with an equivalent function on pNanh_F35 (Nanh_0082, Nanh_0080) is indicative for the mobilization of the smallest plasmid. The RepA-I type replicon pNanh_C61 contains a complete rhamnose operon [66] and it is dominated by genes that are required for polysaccharide biosynthesis. Finally, the presence of CRISPRs (Clustered Regularly Interspaced Short Palindromic Repeats) that provide acquired resistance against viruses [67] on the 56 kb replicon pNanh_E56 is noticeable. The circularity of this scaffold has not been validated experimentally, but the proven circularity of the chromosome supports the localization of these CRISPRs on a plasmid.

**Table 7:** Integrated Microbial Genome (IMG) locus tags of *L. nanhaiensis* DSM 24252^T^ genes^†^

**Replicon**	**Replication initiation**		**Plasmid stability **		**Type IV Secretion**	
	Replicase	Locus tag	Toxin	Antitoxin	VirB4	VirD4
Chromosome	DnaA	Nanh_3012	-	-	-	Nanh_3787^#^
pNanh_A236	RepC-5	Nanh_4695	Nanh_4700	Nanh_4699	Nanh_4884	Nanh_4897^#^
pNanh_B92	DnaA-like I RepA-IVc*	Nanh_0132 Nanh_0112	-	-	-	-
pNanh_C61	RepA-I	Nanh_4577	-	-	-	-
pNanh_D58	RepC-9	Nanh_4622	-	-	-	-
pNanh_E56	RepA-IVb*	Nanh_0004	-	-	-	-
pNanh_F35	RepA-IVa*	Nanh_0078	-	-	-	Nanh_0080^#^

^†^Genes for the initiation of replication, toxin/antitoxin modules and two representatives of type IV secretion systems (T4SS) that are required for conjugation.

*solitary replicase without partitioning module

#presence of adjacent DNA relaxase VirD2

## Emended description of *Leisingera nanhaiensis* DSM 24252^T^ Sun *et al*. 2010

*Leisingera nanhaiensis* (nan.hai.en´sis. N.L. fem. adj. *nanhaiensis* referring to Nanhai, the Chinese name for the South China Sea, from where the type strain was isolated).

The description is the same as given by Sun *et al*. [2] with the following modification: The relationship to oxygen of *Leisingera nanhaiensis* DSM 24252^T^ is changed from aerobic to facultatively anaerobic.

## References

[1] Schaefer JK Goodwin KD McDonald IR Murrell JC Oremland RS *Leisingera methylohalidivorans* gen. nov., sp. nov., a marine methylotroph that grows on methyl bromide. Int J Syst Evol Microbiol 2002; 52:851-859 10.1099/ijs.0.01960-0 12054249

[2] Sun F Wang B Liu X Lai Q Du Y Li G Luo J Shao Z *Leisingera nanhaiensis* sp. nov., isolated from marine sediment. Int J Syst Evol Microbiol 2010; 60:275-280 10.1099/ijs.0.010439-0 19651744

[3] Vandecandelaere I Segaert E Mollica A Faimali M Vandamme P. *Leisingera aquimarina* sp. nov., isolated from a marine electroactive biofilm, and emended descriptions of *Leisingera methylohalidivorans* Schaefer *et al* 2002, *Phaeobacter daeponensis* Yoon *et al* 2007 and *Phaeobacter inhibens* Martens *et al* 2006. Int J Syst Evol Microbiol 2008; 58:2788-2793 10.1099/ijs.0.65844-0 19060059

[4] Chen Y Comparative genomics of methylated amine utilization by marine *Roseobacter* clade bacteria and development of functional gene markers (*tmm, gmaS*). Environ Microbiol 2012; 14:2308-2322 10.1111/j.1462-2920.2012.02765.x 22540311

[5] Göker M Cleland D Saunders E Lapidus A Nolan M Lucas S Hammon N Deshpande S Cheng JF Tapia R Complete genome sequence of *Isosphaera pallida* type strain (IS1B^T^). Stand Genomic Sci 2011; 4:63-71 10.4056/sigs.1533840 21475588 PMC3072084

[6] Brinkhoff T Bach G Heidorn T Liang LF Schlingloff A Simon M Antibiotic production by a *Roseobacter* clade-affiliated species from the German Wadden Sea and its antagonistic effects on indigenous isolates. Appl Environ Microbiol 2004; 70:2560-2565 10.1128/AEM.70.4.2560-2565.2003 15066861 PMC383154

[7] Gu J Guo B Wang YN Yu SL Inamori R Qu R Ye YG Wu XL *Oceanicola nanhaiensis* sp. nov., isolated from sediments of the South China Sea. Int J Syst Evol Microbiol 2007; 57:157-160 10.1099/ijs.0.64532-0 17220459

[8] Lin KY Sheu SY Chang PS Cho JC Chen WM *Oceanicola marinus* sp. nov., a marine alphaproteobacterium isolated from seawater collected off Taiwan. Int J Syst Evol Microbiol 2007; 57:1625-1629 10.1099/ijs.0.65020-0 17625206

[9] Ruiz-Ponte C Cilia V Lambert C Nicolas JL *Roseobacter gallaeciensis* sp. nov., a new marine bacterium isolated from rearings and collectors of the scallop *Pecten maximus.* Int J Syst Bacteriol 1998; 48:537-542 10.1099/00207713-48-2-537 9731295

[10] Thrash JC Cho JC Vergin KL Giovannoni SJ Genome sequences of *Oceanicola granulosus* HTCC2516^T^ and *Oceanicola batsensis* HTCC2597^T^. J Bacteriol 2010; 192:3549-3550 10.1128/JB.00412-10 20418400 PMC2897662

[11] Vandecandelaere I Segaert E Mollica A Faimali M Vandamme P *Phaeobacter caeruleus* sp. nov., a blue-coloured, colony-forming bacterium isolated from a marine electroactive biofilm. Int J Syst Evol Microbiol 2009; 59:1209-1214 10.1099/ijs.0.002642-0 19406821

[12] Yoon JH Kang SJ Lee SY Oh KH Oh TK *Seohaeicola saemankumensis* gen. nov., sp. nov., isolated from a tidal flat. Int J Syst Evol Microbiol 2009; 59:2675-2679 10.1099/ijs.0.011312-0 19625441

[13] Yoon JH Kang SJ Lee SY Oh TK *Phaeobacter daeponensis* sp. nov., isolated from a tidal flat of the Yellow Sea in Korea. Int J Syst Evol Microbiol 2007; 57:856-861 10.1099/ijs.0.64779-0 17392219

[14] Yuan J Lai Q Wang B Sun F Liu X Du Y Li G Gu L Zheng T Shao Z *Oceanicola pacificus* sp. nov., isolated from a deep-sea pyrene-degrading consortium. Int J Syst Evol Microbiol 2009; 59:1158-1161 10.1099/ijs.0.003400-0 19406811

[15] Zhang DC Li HR Xin YH Liu HC Chi ZM Zhou PJ Yu Y *Phaeobacter arcticus* sp. nov., a psychrophilic bacterium isolated from the Arctic. Int J Syst Evol Microbiol 2008; 58:1384-1387 10.1099/ijs.0.65708-0 18523182

[16] Zheng Q Chen C Wang YN Jiao N *Oceanicola nitratireducens* sp. nov., a marine alphaproteobacterium isolated from the South China Sea. Int J Syst Evol Microbiol 2010; 60:1655-1659 10.1099/ijs.0.016311-0 19717582

[17] Pagani I Liolios K Jansson J Chen IM Smirnova T Nosrat B Markowitz VM Kyrpides NC The Genomes OnLine Database (GOLD) v.4: status of genomic and metagenomic projects and their associated metadata. Nucleic Acids Res 2012; 40:D571-D579 10.1093/nar/gkr1100 22135293 PMC3245063

[18] Riedel T Teshima H Petersen J Fiebig A Davenport K Dalingault H Erkkila T Gu W Munk C Xu Y Genome sequence of the *Leisingera aquimarina* type strain (DSM 24565^T^), a member of the *Roseobacter*clade rich in extrachromosomal elements. Stand Genomic Sci 2013; 8:389-402 10.4056/sigs.3858183 24501625 PMC3910692

[19] Beyersmann PG Chertkov O Petersen J Fiebig A Chen A Pati A Ivanova N Lapidus A Goodwin LA Chain P Genome sequence of *Phaeobacter caeruleus* type strain (DSM 24564^T^), a surface-associated member of the marine *Roseobacter* clade. Stand Genomic Sci 2013; 8:403-419 10.4056/sigs.3927626 24501626 PMC3910702

[20] Freese H Dalingault H Petersen J Pradella S Fiebig A Davenport K Teshima H Chen A Pati A Ivanova N Genome sequence of the plasmid and phage-gene rich marine *Phaeobacter arcticus* type strain (DSM 23566^T^). Stand Genomic Sci 2013; 8:450-464 10.4056/sigs.383362 24501630 PMC3910698

[21] Dogs M Voget S Teshima H Petersen J Fiebig A Davenport K Dalingault H Chen A Pati A Ivanova N Genome sequence of *Phaeobacter inhibens* strain T5^T^, a secondary-metabolite producing member of the marine *Roseobacter* clade, and emendation of the species *Phaeobacter inhibens*. Stand Genomic Sci 2013; 9:334-350 10.4056/sigs.4448212 24976890 PMC4062626

[22] Buddruhs N Chertkov O Fiebig A Petersen J Chen A Pati A Ivanova N Lapidus A Goodwin LA Chain P Complete genome sequence of the marine methyl-halide oxidizing *Leisingera methylohalidivorans* type strain (DSM 14336^T^), a member of the *Roseobacter* clade. Stand Genomic Sci 2013; 9:128-141 10.4056/sigs.4297965 24501651 PMC3910543

[23] Dogs M Teshima H Petersen J Fiebig A Chertkov O Dalingault H Chen A Pati A Goodwin LA Chain P Genome sequence of *Phaeobacter daeponensis* type strain (DSM 23529^T^), a facultatively anaerobic bacterium isolated from marine sediment, and emendation of *Phaeobacter daeponensis*. Stand Genomic Sci 2013; (In press). 10.4056/sigs.4287962 PMC3910554 24501652

[24] Field D Garrity G Gray T Morrison N Selengut J Sterk P Tatusova T Thomson N Allen MJ Angiuoli SV The minimum information about a genome sequence (MIGS) specification. Nat Biotechnol 2008; 26:541-547 10.1038/nbt1360 18464787 PMC2409278

[25] Field D Amaral-Zettler L Cochrane G Cole JR Dawyndt P Garrity GM Gilbert J Glöckner FO Hirschman L Karsch-Mzrachi I PLoS Biol 2011; 9:e1001088 10.1371/journal.pbio.1001088 21713030 PMC3119656

[26] Woese CR Kandler O Wheelis ML Towards a natural system of organisms: proposal for the domains *Archaea*, *Bacteria*, and *Eucarya.* Proc Natl Acad Sci USA 1990; 87:4576-4579 10.1073/pnas.87.12.4576 2112744 PMC54159

[27] Garrity GM, Bell JA, Lilburn T. Phylum XIV. *Proteobacteria* phyl. nov. *In*: DJ Brenner, NR Krieg, JT Staley, GM Garrity (eds), Bergey's Manual of Systematic Bacteriology, second edition, vol. 2 (The *Proteobacteria*), part B (The *Gammaproteobacteria*), Springer, New York, 2005, p. 1.

[28] Validation List No 107. List of new names and new combinations previously effectively, but not validly, published. Int J Syst Evol Microbiol 2006; 56:1-6 10.1099/ijs.0.64188-0 16403855

[29] Garrity GM, Bell JA, Lilburn T. Class I. *Alphaproteobacteria* class. nov. *In*: Garrity GM, Brenner DJ, Krieg NR, Staley JT (eds), Bergey's Manual of Systematic Bacteriology, Second Edition, Volume 2, Part C, Springer, New York, 2005, p. 1.

[30] Garrity GM, Bell JA, Lilburn T. Order III. *Rhodobacterales* ord. nov. *In*: Garrity GM, Brenner DJ, Krieg NR, Staley JT (eds), Bergey's Manual of Systematic Bacteriology, Second Edition, Volume 2, Part C, Springer, New York, 2005, p. 161.

[31] Garrity GM, Bell JA, Lilburn T. Family I. *Rhodobacteraceae* fam. nov. In: Garrity GM, Brenner DJ, Krieg NR, Staley JT (eds), Bergey's Manual of Systematic Bacteriology, Second Edition, Volume 2, Part C, Springer, New York, 2005, p. 161.

[32] Martens T Heidorn T Pukall R Simon M Tindall BJ Brinkhoff T Reclassification of *Roseobacter gallaeciensis* Ruiz-Ponte et al. 1998 as *Phaeobacter gallaeciensis* gen. nov., comb. nov., description of *Phaeobacter inhibens* sp. nov., reclassification of *Ruegeria algicola* (Lafay et al. 1995) Uchino et al. 1999 as *Marinovum algicola* gen. nov., comb. nov., and emended descriptions of the genera *Roseobacter*, *Ruegeria* and *Leisingera* Int J Syst Evol Microbiol 2006; 56:1293-1304 10.1099/ijs.0.63724-0 16738106

[33] http://www.baua.de/.

[34] Ashburner M Ball CA Blake JA Botstein D Butler H Cherry JM Davis AP Dolinski K Dwight SS Eppig JT Gene ontology: tool for the unification of biology. The Gene Ontology Consortium. Nat Genet 2000; 25:25-29 10.1038/75556 10802651 PMC3037419

[35] Mavromatis K Land ML Brettin TS Quest DJ Copeland A Clum A Goodwin L Woyke T Lapidus A Klenk HP The fast changing landscape of sequencing technologies and their impact on microbial genome assemblies and annotation. PLoS ONE 2012; 7:e48837 10.1371/journal.pone.0048837 23251337 PMC3520994

[36] List of growth media used at DSMZ: http://www.dsmz.de/catalogues/catalogue-microorganisms/culture-technology/list-of-media-for-microorganisms.html

[37] Gemeinholzer B Dröge G Zetzsche H Haszprunar G Klenk HP Güntsch A Berendsohn WG Wägele JW The DNA Bank Network: the start from a German initiative. Biopreserv Biobank 2011; 9:51-55 10.1089/bio.2010.0029 24850206

[38] Bennett S Solexa Ltd. Pharmacogenomics 2004; 5:433-438 10.1517/14622416.5.4.433 15165179

[39] The DOE Joint Genome Institute http://www.jgi.doe.gov/

[40] Zerbino D Birney E Velvet: Algorithms for de novo short read assembly using de Bruijn graphs. Genome Res 2008; 18:821-829 10.1101/gr.074492.107 18349386 PMC2336801

[41] Butler J MacCallum I Kleber M Shlyakhter IA Belmonte MK Lander ES Nusbaum C Jaffe DB ALLPATHS: de novo assembly of whole-genome shotgun microreads. Genome Res 2008; 18:810-820 10.1101/gr.7337908 18340039 PMC2336810

[42] Ewing B Green P Base-calling of automated sequencer traces using phred. II. Error probabilities. Genome Res 1998; 8:186-194 10.1101/gr.8.3.175 9521922

[43] Ewing B Hillier L Wendl MC Green P Base-calling of automated sequencer traces using phred. I. Accuracy assessment. Genome Res 1998; 8:175-185 10.1101/gr.8.3.175 9521921

[44] Gordon D Abajian C Green P Consed: a graphical tool for sequence finishing. Genome Res 1998; 8:195-202 10.1101/gr.8.3.195 9521923

[45] Hyatt D Chen GL LoCascio PF Land ML Larimer FW Hauser LJ Prodigal: prokaryotic gene recognition and translation initiation site identification. BMC Bioinformatics 2010; 11:119 10.1186/1471-2105-11-119 20211023 PMC2848648

[46] Mavromatis K Ivanova NN Chen IM Szeto E Markowitz VM Kyrpides NC The DOE-JGI Standard operating procedure for the annotations of microbial genomes. Stand Genomic Sci 2009; 1:63-67 10.4056/sigs.632 21304638 PMC3035208

[47] Pati A Ivanova NN Mikhailova N Ovchinnikova G Hooper SD Lykidis A Kyrpides NC GenePRIMP: a gene prediction improvement pipeline for prokaryotic genomes. Nat Methods 2010; 7:455-457 10.1038/nmeth.1457 20436475

[48] Markowitz VM Ivanova NN Chen IMA Chu K Kyrpides NC IMG ER: a system for microbial genome annotation expert review and curation. Bioinformatics 2009; 25:2271-2278 10.1093/bioinformatics/btp393 19561336

[49] del Solar G Giraldo R Ruiz-Echevarria MJ Espinosa M Diaz-Orejes R Replication and control of circular bacterial plasmids. Microbiol Mol Biol Rev 1998; 62:434-464 9618448 10.1128/mmbr.62.2.434-464.1998 PMC98921

[50] Petersen J Phylogeny and compatibility: plasmid classification in the genomics era. Arch Microbiol 2011; 193:313-321 21374058 10.1007/s00203-011-0686-9

[51] Chen Y Patel NA Crombie A Scrivens JH Murrell C Bacterial flavin-containing monooxygenase is trimethylamine monooxygenase. Proc Natl Acad Sci USA 2011; 108:17791-17796 10.1073/pnas.1112928108 22006322 PMC3203794

[52] Lang AS Beatty JT The gene transfer agent of *Rhodobacter capsulatus* and “constitutive transduction” in prokaryotes. Arch Microbiol 2001; 175:241-249 10.1007/s002030100260 11382219

[53] Thole S Kalhoefer D Voget S Berger M Engelhardt T Liesegang H Wollherr A Kjelleberg S Daniel R Simon M *Phaeobacter gallaeciensis* genomes from globally opposite locations reveal high similarity of adaption to surface life. ISME J 2012; 6:2229-2244 10.1038/ismej.2012.62 22717884 PMC3504968

[54] Buddruhs N Pradella S Göker M Päuker O Michael V Pukall R Spröer C Schumann P Petersen J Brinkhoff T Molecular and phenotypic analyses reveal the non-identity of the *Phaeobacter gallaeciensis* type strain deposits CIP 105210^T^ and DSM 17395. Int J Syst Evol Microbiol 2013; 63:4340-4349 10.1099/ijs.0.053900-0 24187021

[55] Cypionka H Pfennig N Growth yields of *Desulfotomaculum orientes* with hydrogen in chemostat culture. Arch Microbiol 1986; 143:396-399 10.1007/BF00412808

[56] Griess JE Bemerkungen zu der Abhandlung der HH. Weselsky und Benedikt „Ueber einige Azoverbindungen”. Ber. Deutsch. Chem. Ges. 1879; 12:426 10.1002/cber.187901201117

[57] Auch AF Klenk HP Göker M Standard operating procedure for calculating genome-to-genome distances based on high-scoring segment pairs. Stand Genomic Sci 2010; 2:142-148 10.4056/sigs.541628 21304686 PMC3035261

[58] Auch AF Von Jan M Klenk HP Göker M Digital DNA-DNA hybridization for microbial species delineation by means of genome-to-genome sequence comparison. Stand Genomic Sci 2010; 2:117-134 10.4056/sigs.531120 21304684 PMC3035253

[59] Meier-Kolthoff JP Auch AF Klenk HP Göker M Genome sequence-based species delimitation with confidence intervals and improved distance functions. BMC Bioinformatics 2013; 14:60 10.1186/1471-2105-14-60 23432962 PMC3665452

[60] Petersen J Phylogeny and compatibility: plasmid classification in the genomics era. Arch Microbiol 2011; 193:313-321 21374058 10.1007/s00203-011-0686-9

[61] Petersen J Brinkmann H Berger M Brinkhoff T Päuker O Pradella S Origin and evolution of a novel DnaA-like plasmid replication type in *Rhodobacterales*. Mol Biol Evol 2011; 28:1229-1240 10.1093/molbev/msq310 21097494

[62] Petersen J Brinkmann H Pradella S Diversity and evolution of repABC type plasmids in *Rhodobacterales*. Environ Microbiol 2009; 11:2627-2638 10.1111/j.1462-2920.2009.01987.x 19601964

[63] Zielenkiewicz U Ceglowski P Mechanisms of plasmid stable maintenance with special focus on plasmid addiction systems. Acta Biochim Pol 2001; 48:1003-1023 11995964

[64] Cascales E Christie PJ The versatile bacterial type IV secretion systems. Nat Rev Microbiol 2003; 1:137-149 10.1038/nrmicro753 15035043 PMC3873781

[65] Petersen J Frank O Göker M Pradella S Extrachromosomal, extraordinary and essential-the plasmids of the *Roseobacter* clade. Appl Microbiol Biotechnol 2013; 97:2805-2815 10.1007/s00253-013-4746-8 23435940

[66] Giraud MF Naismith JH The rhamnose pathway. Curr Opin Struct Biol 2000; 10:687-696 10.1016/S0959-440X(00)00145-7 11114506

[67] Barrangou R Fremaux C Deveau H Richards M Boyaval P Moineau S Romero DA Horvath P CRISPR provides acquired resistance against viruses in prokaryotes. Science 2007; 315:1709-1712 10.1126/science.1138140 17379808

